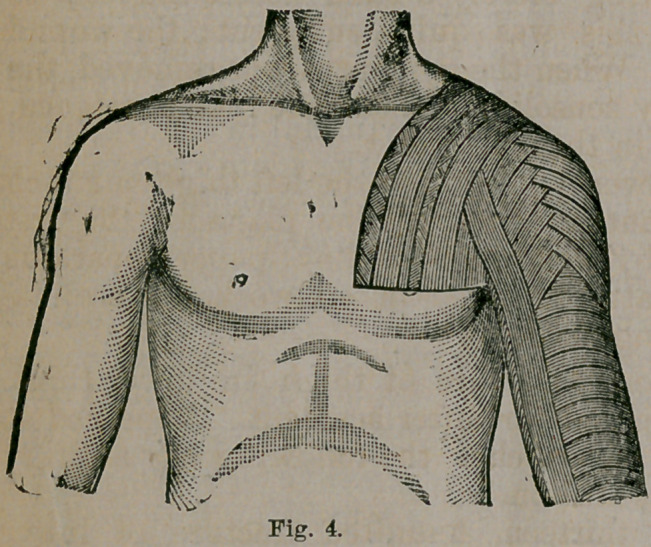# Manila Paper, as a Material for Splints and the Immovable Apparatus

**Published:** 1871-04

**Authors:** R. O. Cowling

**Affiliations:** Demonstrator of Anatomy in the University of Louisville


### MANILA PAPER AS A MATERIAL FOR SPLINTS AND THE IMMOVABLE APPARATUS.

BY R. O. COWLING, M. D., Demonstrator of Anatomy in the University of Louisville.

An apology seems necessary for introducing a new material for splints, The list is crowded with representatives from every kingdom of nature, and the bones themselves have been used for this purpose; as, for example, by Baron Larrey in the Egyptian campaign. The material, however, now offered has so many excellencies that it is believed a trial of it will establish its superiority to all others in very many cases. It is conveniently and cheaply obtained, easily adapted, and almost universally applicable; and, what must have been overlooked by the profession, when starch possesses a firmness inferior only to metal or plaster; while its elasticity gives it an immense advantage over the last named substance when used for the immovable apparatus. In fractures of the femur, wherever situated, when using the immovable apparatus, I have had difficulty in confining the hip-joint. When the bone has been broken high up, the difficulty of supporting the upper fragment is added. In these cases, the ordinary spica is objectionable on several accounts. If a sufficient number of layers of the roller are used to give it a proper firmness, the accumulation of folds in the perinæum is a source of discomfort; while the abdominal turns, in addition to this, soon become loosened, and interfere with the security of the hip. The pasteboard splints, which it has been recommended to carry higher up to re-inforce the spica, are not readily molded. It was in this connection that I hit upon the use of paper. In the experiments first made on a wooden model, ordinary newspaper was the variety used; but while this made an admirable mold, too many layers were required to give it firmness.

Manila paper was then substituted, and is the variety I have since used, not only to make the spica, but the entire apparatus. This paper, made from manila hemp, sustains a great degree of tension, even when wet with the stiffening material. It is the kind used for cartridges, paper bags, etc., and comes in various thicknesses. The thicker varieties are best adapted for the purposes now considered. In its absence, any thick paper may be conveniently substituted. The method of applying it in the immovable apparatus is illustrated in Fig. 1. The paper is cut into slips from half an inch to two inches in breadth, and of a sufficient

Fig. 1.

length. The limb is enveloped in cotton, and the paper is starched and put on in circular folds, as with the many-tailed bandage. Longitudinal and circular layers are then added. It will be found more convenient to confine the cotton with a dry roller. While the application in this way is far more tedious, the apparatus may afterward be more easily divided. The narrower slips are applied over the foot, and the broader only where the irregularities of the limb are slight; otherwise, the paper will not adapt itself well. On reaching the crotch, cover in the hip with oblique turns, passing alternately from either side. By this means it, as well as the buttock, may be enveloped without an accumulation of material at any one point. Three layers, representing six thicknesses of paper—as each slip should overlap its neighbor one half—will give sufficient firmness, except over the seat of fracture and the contiguous joints. These points should be reinforced by additional layers. Lastly, a spica bandage should be thrown around the hip until the apparatus dries; afterward, and for greater security, a single band may be left to confine it to the opposite hip. Such is the use of the paper in the immovable apparatus.

Fig. 2.

It remains to be seen wherein it is superior to that made by rollers and splints. If beauty be a recommendation, it certainly possesses that. Its surface is smooth, and the exact contour of the limb is preserved. It is not easily soiled, and when divided its edges remain for weeks or months without fraying. Its firmness far exceeds one made with equal thickness of cloth and splints. It is this point we believe that has been overlooked by the profession. Six or eight thicknesses of the paper give such solidity that seemingly a nut could be cracked over the seat of fracture without disturbance. The models we have made could be bent only with extreme difficulty. It is the old principle of the arch and the tube. No such firmness can be obtained from the cloth alone without the use of splints. Where lightness, then, is sought, it has the advantage. Its cost is almost nothing; a dime's worth of paper will do for a lower extremity of ordinary size. It allows of earlier and better division. Put on with the egg and flour mixture, I have found the paper sufficiently firm in four hours to be easily cut, though it does not attain its full stiffness for a day or two. When ordinary paste is used, the dressing dries more slowly. Fenestra of any size may be cleanly cut, and will retain their shape for months.

If the apparatus be divided, as indicated in Fig. 2, both before and behind, taking the precaution to break joints of apparatus over the seat of fracture, an objection which exists in the minds of many surgeons to the starched apparatus is obviated. Tho two splints thus made may be removed and reapplied without disturbing the limb. Mr. Tuffnell recommends splints for the leg made by longitudinal layers of cloth lined with lint, and praises their accuracy and lightness. I doubt, however, if these splints could be used on the thigh and hip, unless reinforced by pasteboard or some other material.

I have found the paper exceedingly useful in the treatment of sprained ankle. After combating the acute symptoms by position, etc., I apply the roller. When the swelling is reduced I put on the paper boot, extending it a short distance above the joint. A boot of this description can easily be made which fits accurately and insures perfect rest, while its lightness and appearance recommend it to the patient; a point which will be appreciated by those who have seen how loath patients with this injury are to submit to the necessary restraint.

By splitting the boot in front, as is shown in Fig. 3, it can be put on and off in a moment by the patient himself. In the several instances in which I have used the boot the results have been very gratifying. In one case a young gentleman fell a distance of twenty-five feet squarely upon his right heel. The concussion of the joint was extreme. On the fifth day the paper boot, well lined with cotton, was put on. The comfort was immediate. He took at once to crutches and resumed his business. He wore the apparatus for about three weeks, and during that time he always felt uneasy when he left it off.

Fig. 3.

My actual experience with paper dressings has been confined to the lower extremity. I have made models on other portions of the body which will induce me to try it elsewhere when an occasion arises; for instance, as a shoulder cap for fractures in the upper portion of the humerus. Fig. 4 is from a model taken from a healthy arm. A piece of thick canton-flannel was first wetted and molded to the shoulder. The strips were then put on in circular longitudinal, and oblique turns, as seen in the figure. A spica was then carried over the paper, and kept there for fifteen minutes, when the mold was found to be of sufficient firmness to retain its shape. It was now dried before the fire, trimmed, and proved to be almost as hard as wood, and admitted of most accurate reädjustment. I have found gutta-percha more difficult to mold, while neither pasteboard nor leather can be applied with anything like the same accuracy. I believe that in fractures of the surgical neck of the humerus, by adopting the broad flanges shown in the figure, covering well the pectoral muscle in front and scapula behind, the muscles producing the displacement can be in a great measure controlled. I also made an excellent mold for the lower jaw, which, along with other experiments, convinced me that the use of paper might be extended to almost any part of the body where molds are called for, as for spinal support, angular splints at the elbow, and bracketed splints for compound fractures.

Fig. 4.

Since I begun using the paper my attention has been called to its previous employment by others. A physician in this city reports the use of newspaper splints by a surgeon in the Confederate army. As described by him, the method consisted in soaking the folded newspapers in starch, applying to the sides of the limb, and compressing with a bandage. This, while perhaps superior in some respects to the method we have described, is still altogether different.

M. Laugier recommended, in 1838, tarred paper, cut in strips, for the immovable apparatus; but he made no mention of other uses to which it might be put, and did not even hint at the *stiff spica*, which constitutes in my eyes one of the chief advantages possessed by the paper. Malgaigne, who quotes M. Laugier, dismisses the subject with the remark that paper might answer where cotton or linen could not be obtained. But my purpose is to ask the attention of the readers of the American Practitioner to what I have found to be really a most admirable substance for bandages and splints, rather than to claim originality in its application.

I have used the manila paper in the following cases:

*First.* A fracture of the femur in its upper fourth, complicated with a compound dislocation of the ankle, in a patient aged fifty; traumatic delirium for two weeks, during which time the straight splints and bandage, the inclined plane and ordinary starched apparatus were used in turn. but which, in his ravings, the patient tore off. It was in this case that I was struck with the lack of power of the ordinary spica in supporting the upper fragment. The paper apparatus was now applied, and from this time the patient certainly had greater ease. A fenestrum about nine inches in length was cut over the injured ankle, taking away half of the dressing at that point, and leaving merely a band across the toes to support the foot; yet this was quite sufficient; the wound closed in nine weeks. When the apparatus was removed, the thigh was found firmly consolidated; but one inch shortened, and motion preserved in the ankle-joint.

*Second.* C. F., age twenty, fractured the left thigh one inch below the great trochanter. Used incline plane for the first week, with extension by weight and pulley; paper apparatus then applied. Apparatus removed in six weeks; consolidation perfect; shortening one inch.*

*Third.* A. C., age forty, fracture of thigh in lower third. Apparatus applied twelve hours after accident. Appeared at Prof. Yandell's clinic on crutches the following day entirely comfortable, apparatus still on.

*Fourth.* H. B., age thirteen, ununited fracture of lower third of femur of three months' standing. Injury had been treated with straight splints and roller. Paper apparatus apparatus applied; union in two weeks.—*American Practitioner.*

LOUISVILLE, DECEMBER.

*Professor D. W. Yandell's case.

## Figures and Tables

**Fig. 1. f1:**
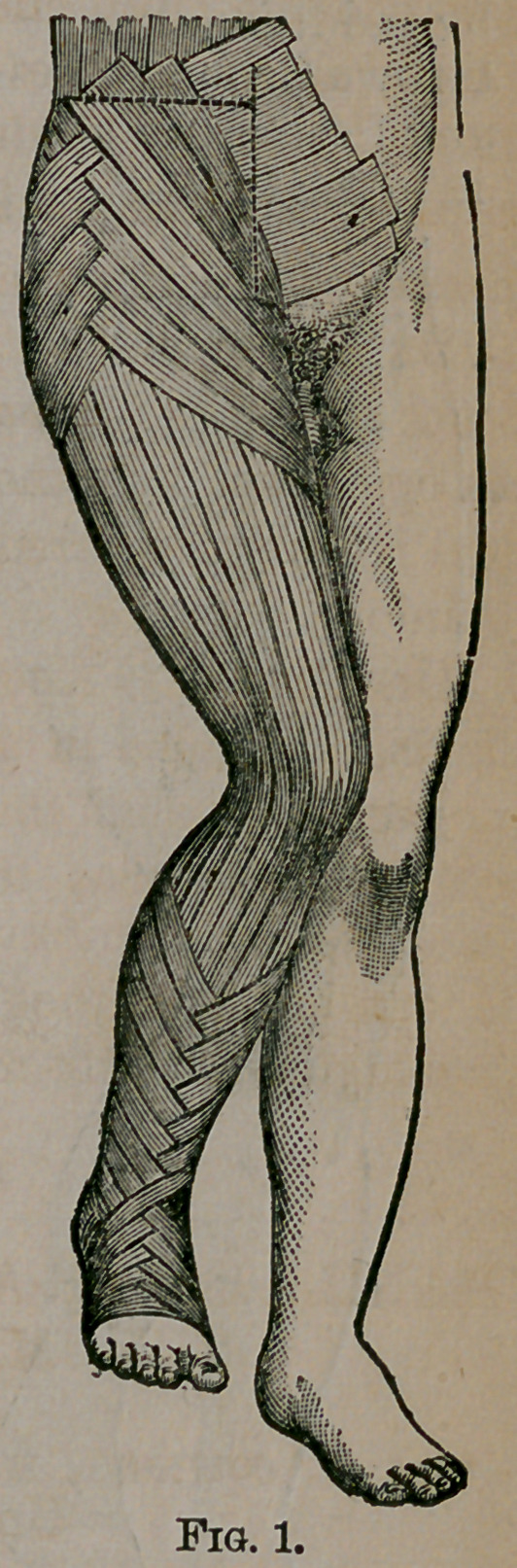


**Fig. 2. f2:**
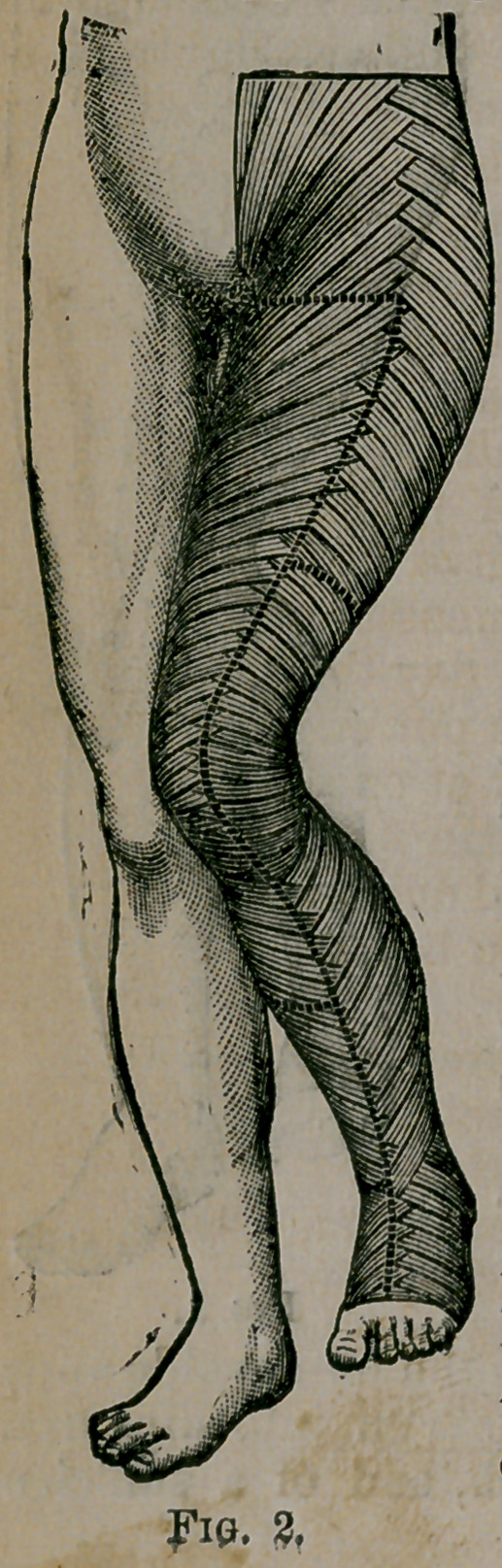


**Fig. 3. f3:**
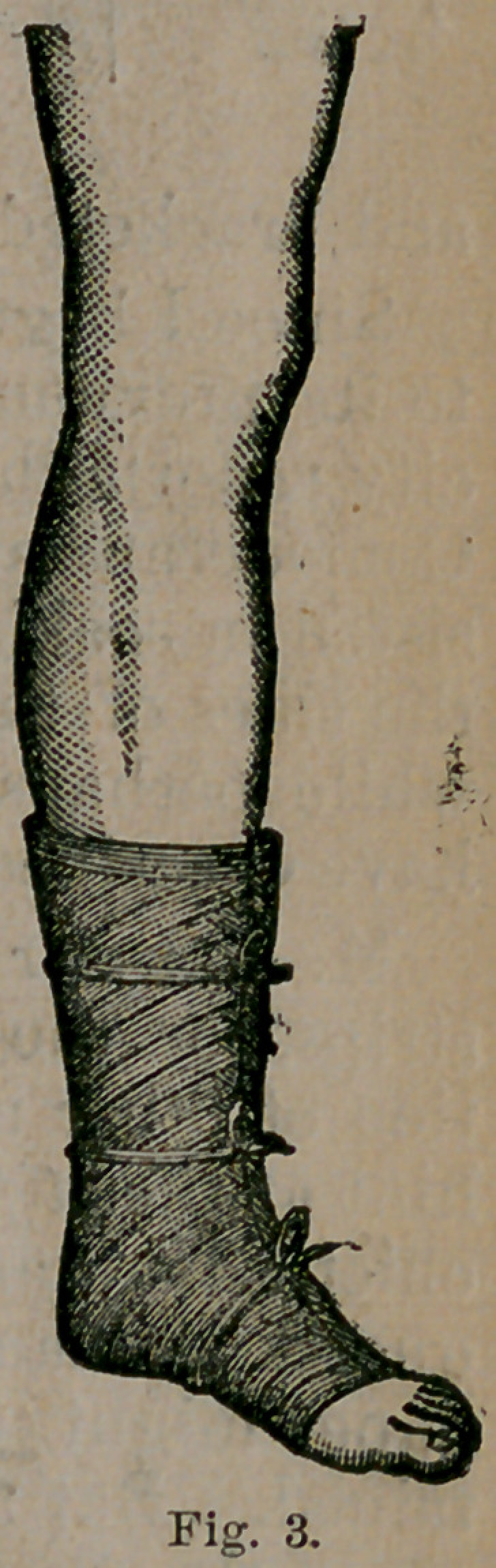


**Fig. 4. f4:**